# Distal Triceps Tendon Rupture—First Retrospective Study in Central Europe

**DOI:** 10.3390/jcm13247792

**Published:** 2024-12-20

**Authors:** Jaroslav Sekáč, Peter Šagát, Peter Bartík, Miroslav Kilián, Dragoş Ioan Tohănean, Jason Perez, Veronika Vasilcova, Štefan Durdík

**Affiliations:** 1 Faculty of Medicine, Comenius University, 81372 Bratislava, Slovakia; jaroslavsekac@yahoo.com; 2 GSD/Health and Physical Education Department, Sport Sciences and Diagnostic Research Group, Prince Sultan University, Riyadh 11586, Saudi Arabia; pbartik@psu.edu.sa (P.B.); vasilcovave@mngha.med.sa (V.V.); 3 Department of Trauma Surgery, Slovak Medical University and University Hospital, 82606 Bratislava, Slovakia; miroslav.kilian@szu.sk (M.K.); jperez@psu.edu.sa (J.P.); 4 Faculty of Physical Education and Mountain Sports, Transilvania University of Brasov, 500019 Braşov, Romania; dragos.tohanean@unitbv.ro; 5 King Abdulaziz Medical City, Riyadh 11426, Saudi Arabia; 6 Clinic of Oncology and Surgery, Faculty of Medecine, Comenius University, 81250 Bratislava, Slovakia; stefan.durdik@ousa.sk

**Keywords:** distal triceps rupture, triceps repair, transosseous, suture anchor, rerupture, range of motion

## Abstract

**Background:** This retrospective study is the only one in the last 10 years from central Europe and provides a current picture of prevalence, new diagnostic modalities, new methods of surgical treatment, and also offers new insights into post-operative care. Triceps tendon rupture is the least reported among all the tendon injuries in the literature. In general, effective treatments for tendon injuries are lacking because the understanding of tendon biology lags behind that of the other components of the musculoskeletal system. Tendon tissue has a low number of cells and growth hormones and thus a lack of natural healing ability. Understanding the links between the mechanical and biological parameters involved in tendon development, homeostasis, and repair is a prerequisite for the identification of effective treatments for chronic and acute tendon injuries. **Methods:** The authors statistically evaluated the set of patients with this diagnosis in the largest University Hospital in Slovakia over the last 10 years. **Results:** Between 2014 and 2023, 23 patients with distal triceps tendon ruptures (DTTR) were treated at University Hospital. In some years not a single patient with this diagnosis underwent surgery, reinforcing the idea that DTTR may be either rare or underdiagnosed. The incidence in our region is 0.46 cases per 100,000 inhabitants. The average age of patients was 57.7 years, with a male predominance of 90%. Less than half of the patients (43.5%) underwent surgical intervention, and the median time from injury to surgery was less than 10 days. This rapid timeline indicates a high standard of medical care, given the semi-elective nature of the surgery and the need for MRI (Magnetic Resonance Imaging) confirmation of tendon rupture exceeding 50% of the fibers before proceeding with surgery. The three standard surgical techniques were employed in approximately equal proportions. **Conclusions:** This study suggests that none of the methods is currently preferred, and that the choice of the technique was largely determined by perioperative findings and the surgeon’s discretion. Post-operative complications were minimal, with only one patient experiencing any issues after surgery.

## 1. Introduction

**Figure 1:**
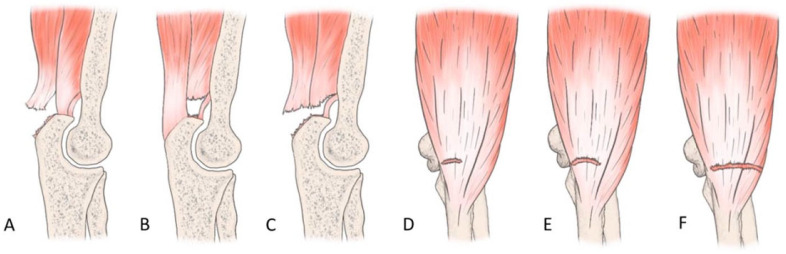
Illustration of the three basic types of DTTR partial rupture. (**A**): “Superficial tear”—the tendinous portion of the lateral and long head of the triceps (**B**): “Deep tear”—the deep muscular portion of the distal triceps involving the medial head of the triceps (**C**): “Full body tear”—a sub complete tear of the DTTR. (**D**–**F**)—coronal planes showing partial, complete, and lateral involvement [10].

**Figure 2:**
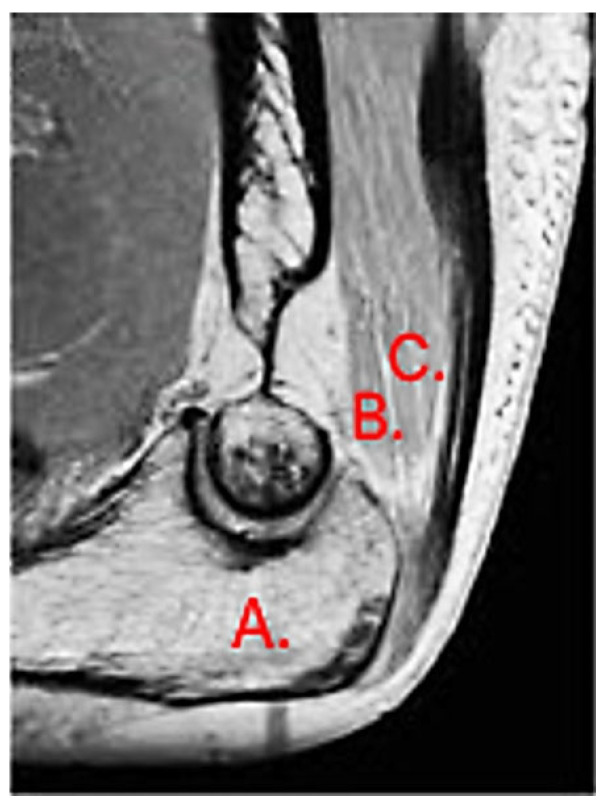
Normal anatomic attachment relationships of the triceps tendon in sagittal section to the olecranon ulnae. MRI: A—olecranon, B—medial head, C—common tendon of lateral and long head (own source).

**Figure 3:**
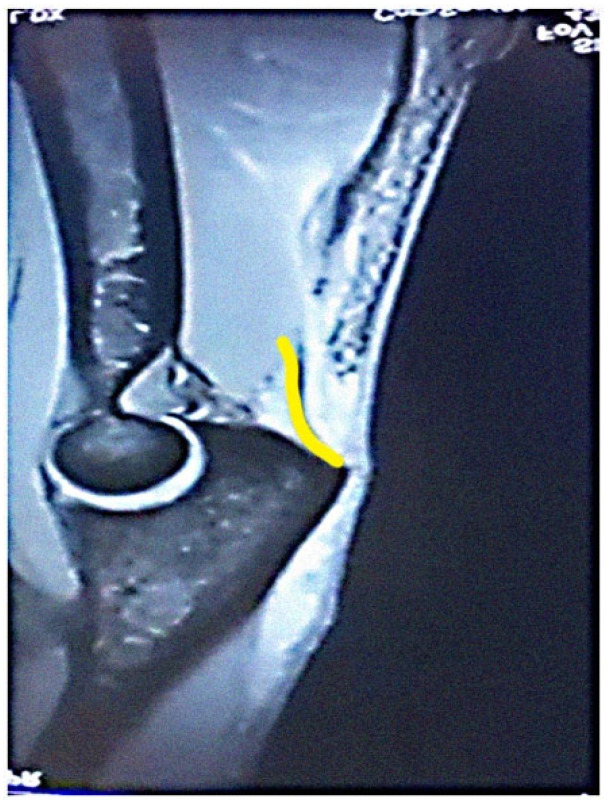
Sub complete rupture of the triceps tendon of the right hand (own source). Yellow line: that is the place of the rupture.

**Figure 4:**
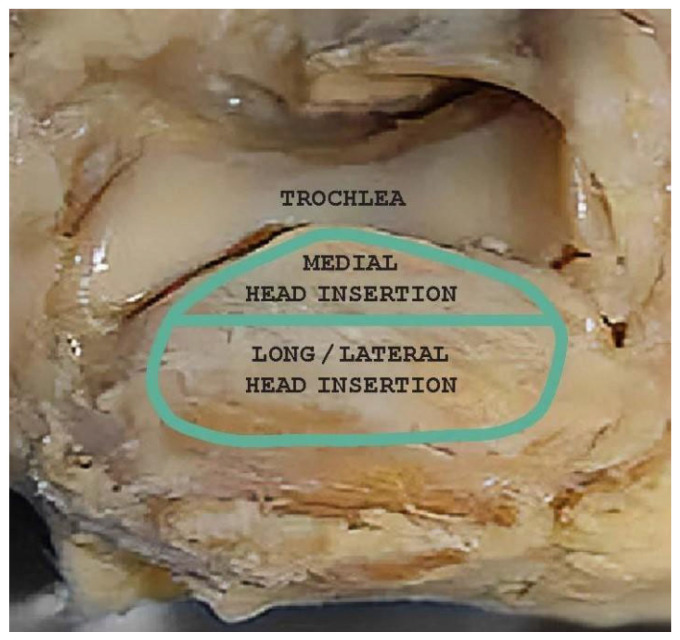
Place of attachment of the individual heads of the triceps to the olecranon ulnae (Own source).

The most common tendon injuries are rotator cuff tears of the shoulder, hand flexor injuries, and achilles tendon injuries [1]. Triceps tendon rupture is the least reported among all the tendon injuries in the literature [2,3]. 65 years ago, Anzel evaluated a series of 1014 patients with tendon ruptures in various locations, and triceps tendon ruptures accounted for only 0.8% of this series [4]. Currently the prevalence is increasing, and the prevalence of triceps tendon injuries has been found to be 3.8% [5]. Theoretically, the types of tendon injuries are tendon avulsion or inside the muscle belly. In practice, a rupture almost always occurs in the area of the tendon-bone junction, and the cause is an eccentric contraction of the triceps causing a tendon deformity of more than 8% [6]. Traditionally the triceps tendon has a uniform attachment to the olecranon ulnae. This premise has caused problems in assessing the degree of damage in traumatic triceps tendon ruptures. In 2006, an anatomic study by Madsen confirmed that in most cases the medial head of the triceps has a single attachment to the olecranon ulnae [7]. This insertion is located in a deeper layer and forms a narrower part of the attachment, and very rarely is only this part damaged [8]. The long and lateral head of the triceps has a common attachment that runs more superficially, gradually extending laterally into the surrounding area towards the musculus anconeus, which helps to strengthen the bone-tendon junction. The width of the attachment correlates with the size of the olecranon and ranges from 20 to 40 mm. Paradoxically, the thickness of the tendon is not as pronounced. The attachment itself occupies a large surface area, reaching 400 mm^2^ in diameter and is dome-shaped [9]. These current findings are particularly important in partial tendon ruptures when a decision has to be made whether to proceed conservatively or with surgical revision. MRI is an appropriate method of choice to accurately assess the current condition. A schematic representation of the three basic types of partial DTTR rupture can be seen in Figure 1, while the normal anatomic attachment relationships of the triceps tendon in sagittal section to the olecranon ulna and a sub-complete rupture of the triceps tendon of the right hand are shown in Figure 2 and Figure 3. Furthermore, the place of attachment of the individual heads of the triceps to the olecranon ulnae is presented in Figure 4.

## 2. Diagnosis and Design of Appropriate Treatment

**Figure 5:**
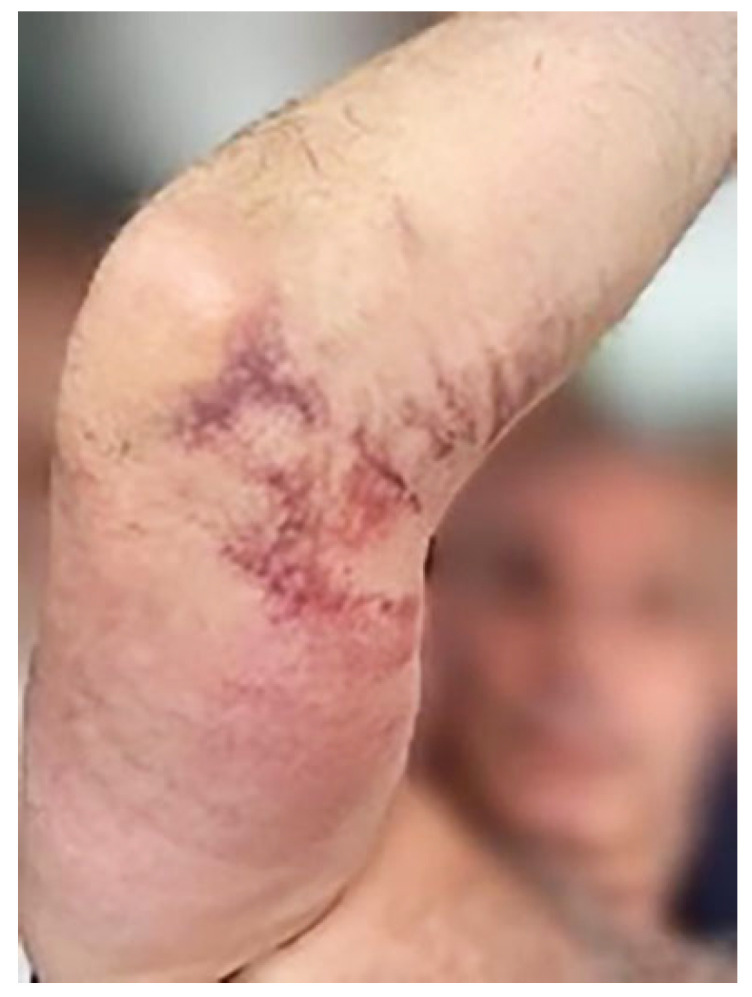
Condition immediately after the injury, mobility in the elbow joint was preserved as it was a partial lesion that gradually expanded with insufficient immobilization. Partial tears may be easily missed, as patients may have a good range of active motion but do typically present with reduced power on extension of the elbow. A palpable “step-off” defect will confirm the diagnosis (own source).

It is surprising, given the ease of access and palpation of the triceps tendon in a healthy individual, how many incorrect diagnostic conclusions are described in the literature for triceps tendon injuries. Patients presenting with partial tears or complete tears may also have only subtle differences in clinical manifestations. This can lead to difficulty in differential diagnosis and therapeutic decision-making. It is not uncommon for a patient to present to a rehabilitation outpatient clinic with a partial tendon lesion that nevertheless meets the criteria for surgical treatment. Surgically treated acute tendon injuries have a treatment success rate of more than 95%; on the contrary, conservative management always results in a functional deficit, especially in young patients working manually, and studies also indicate an increased risk of re-injury (re-rupture) with a non-operative approach [11]. If more than 50% of the tendon bundles are ruptured and the time since the injury is no more than a few weeks, it is appropriate to refer the patient immediately to an orthopaedic outpatient clinic for consideration of suture and fixation of the tendon to the olecranon ulnae. The examination of the patient begins with taking a medical history with a focus on the mechanism of injury. A fall on an outstretched hand may result in an injury mostly to the lateral and long head of the distal triceps tendon and an intact medial head tendon; however, direct injuries can involve full-thickness ruptures. Clinical examination of the patient alone may not always confirm the diagnosis. Patients typically present with pain, bruising, ecchymosis, swelling, and a decreased active range of motion (Figure 5). Palpable defects are commonly found and present in up to 80% of patients [12]. Provocative tests may be helpful, e.g., the modified Thompson squeeze test: the patient lies prone with the elbow at the end of the table and the forearm hanging down; the triceps muscle is firmly squeezed; and the inability to extend the elbow against gravity suggests complete disruption of the triceps proper and lateral expansion. Incorrect assessment of the extent of the damage results in non-indication of surgical treatment. The second possible scenario points to the risk that more extensive tendon damage (more than 50%) develops into complete damage, and subsequent complete rupture of a chronic nature requires a different surgical procedure. Primary repair of a torn triceps tendon is possible within the first 3 weeks after injury. The management of chronic tendon ruptures is a challenge. Plain radiographs should be presented in every case. The presence of small flakes superior to the olecranon is a tell-tale sign of an avulsion fracture—this picture may not always be present, but on the other hand, its presence confirms the diagnosis. An ultrasonography (US) examination is advantageous in terms of time, cost-effectiveness, and accessibility [13]. Direct patient interaction allows direct correlation with the site of pain through comparison with the contralateral elbow. Dynamic US allows the use of stress maneuvers and visualization of transient conditions that may not otherwise be revealed during static examination. MRI is the method of choice in the indication of surgical treatment for partial tendon lesions. An MRI best evaluates isolated medial head attachment. We then proceed in a non-sodial fashion when evaluating the MRI. On sagittal sections we focus on the olecranon ulnae and see the fat pad overlying it, usually. Next, we look for a hematoma, which confirms that this is a traumatic event. The medial head of the triceps bypasses the fat pad. Its partial rupture is called a deep triceps tendon rupture. Conversely, the remaining two heads of the triceps, when ruptured, are seen retracted several centimeters from the olecranon, superficially deposited and possibly with a small bony fragment detached from the tendon portion of the olecranon. Bursitis, or chronic tendinopathy, is also a frequent concomitant finding.

## 3. Surgical Treatment

In general, any tear that exceeds 50% of the tendon integrity and full-thickness tears are surgically treated. There is consensus that surgical treatment leads to excellent results. Conversely, conservative management can lead to functional deficits, especially in young, active patients performing manual occupations, and studies indicate an increased risk of re-injury (re-tear) with a non-operative approach. There is also a risk of misdiagnostic assessment of the degree of tendon damage in that delayed reconstruction may require a tendon graft. Surgical treatment of the ruptured triceps tendon is currently performed in a day surgery setting. The surgery itself can be performed openly, arthroscopically, or percutaneously. In Slovakia, only the open approach is currently used. Safety at the suture-tendon interface is a critical component of soft tissue fixation. The Krackow suture became a concept after its introduction by Ken Krackow in 1986. Over the next 3 decades, different variants of the Krackow suture technique were developed: the McKeon double Krackow suture (MDK), the Ostrander modified Krackow suture (OMK), and the Wilson double Krackow suture (WDK). Biomechanical testing demonstrated that the MDK had less elongation after cyclic loading than the WDK suture, but no differences in the ultimate load to failure were found between the three groups. No differences in cross-sectional diameter were found between the constructs [14]. The requirements for suture material include good sliding properties, lack of bulkiness, and high tensile strength to maintain tissue approximation. New suture materials with a salt-infused silicone core were designed to minimize laxity and preserve consistent tissue approximation in order to avoid gap formation [15]. Salt attracting the water in a liquid environment and fluid absorption leads to radial expansion of the suture material due to swelling of its core, resulting in shortening of the braid and thus a self-tensioning of the suture material. Also, an important part of surgical technique in tendon suture is the question of how many knots are required to ensure long-term strength in postoperative rehabilitation. The practice of 7 needed knots to achieve knot security is standard for commonly used sewing material [16]. On the other hand, slippage and self-seating of the knots under load are unavoidable even with the highest tying loads. Surgery is preferably performed within the first three weeks of injury. Because of the heterogeneity of types of tears, repair techniques, and outcome measures, it is impossible to determine the superiority of one technique over another. Three basic methods of attaching the tendon to the olecranon are transosseous tunnel only (TT), suture anchor only (SA), and transosseous tunnel plus suture anchor (TTSA) repair techniques. However, given the similarities between the various methods of repair, surgeons can be confident in repairing this type of injury by whichever modality they deem appropriate. Previous data may have encouraged surgeons to perform a more costly procedure in hopes of improved postoperative outcomes. Currently, based on literature sources and our experience, surgical repair of the avulsed tendon has proven to give universally good results irrespective of the technique. There is no consensus among surgeons on the best method of repair. Our surgical technique provides a solid tendon repair without the need for further osteosynthetic removal. Despite the success of operative repair, it is also associated with a relatively high complication rate, with reports as high as 22%. Common complications are re-rupture, infection, and ulnar neuropathy. For chronic ruptures, a primary tendon repair is not possible due to the degenerated tendon quality with significant tendon retraction, and tendon transfers are required. The reconstruction with a graft includes achilles allograft or ipsilateral semitendinous tendon, anconeus, latissimus dorsi, plantaris, or palmaris longus tendon. Partial tears can be treated conservatively with bracing and physio.

### Post-Operative Course—Phases of the Healing Process

There is no consensus in the literature regarding the optimal postoperative protocol, the duration of immobilization, and the timing of rehabilitation. We recommend the rehabilitation program be divided into three phases. Common sense is the rule. When setting up the protocol, it is advisable if the physiotherapist has the hospital discharge report and the operative protocol. Age, associated conditions, preoperative extent of tendon damage, and method of tendon fixation are taken into account, and an individual rehabilitation plan is created [17]. One-size-fits-all does not exist. The healing process in the case of a ruptured tendon takes place in three phases [18]. Understanding the healing process of the tendon is the pathway to successful restoration of function.

## 4. Phase 1

### 4.1. Histopathologist’s View: Phase 1—Inflammation

Every healing process begins with an inflammatory reaction [19]. Initially, a fibrin network is formed at the site of injury, which facilitates the migration of macrophages and neutrophils to the site of injury [20]. Wound macrophages are activated with the help of rPDGF-BB (recombinant platelet-derived growth factor-BB) [21]. We have two main phenotypes—M1 macrophages, which have a pro-inflammatory effect, creating a protective barrier against pathogens and removing waste products of metabolism. The other type is M2 macrophages, which promote healing and tissue remodeling by releasing specific growth factors [1,20,22,23]. At the same time, pro-inflammatory cytokines and other substances are released from these cells to aid the healing process. Another important component is the tenocytes that arrives at the site of damage. Tenocytes are the cells in which the extracellular matrix (ECM) and its most important component, collagen, and growth factors are formed [24]. The inflammation phase lasts only a few days after the injury (or surgery) and ends around the time when the sutures are removed from the surgical wound and the first follow-up by the surgeon is carried out, i.e., week 2. We can also stimulate this phase medially or, on the other hand, dampen it. As an example, Virchenko’s findings that systemic administration of non-steroidal anti-inflammatory drugs (NSAIDs) for 7 days after achilles tendon injury in rats produced tendon healing with lower mechanical resistance and reduced cross-sectional area of the healed area [25]. On the other hand, tendon healing was improved when the drug was started only from day 6 post-operatively, which is explained by the cessation of excessive pro-inflammatory activity [26]. Therefore, the early inflammatory cascade is neither to be disrupted nor dampened and is necessary to restore the native properties of the tendon. Whereas in the later period, the persistence of inflammatory activity has, on the contrary, a detrimental effect on healing.

### 4.2. Rehabilitation Physician’s View: Phase 1—Protective

Early mobilization after tendon surgery is crucial to avoid commonly observed post-operative soft tissue adhesions. Today, an individually manufactured dynamic brace is applied directly in the operating room with adjustment of the possible passive flexion according to the extent of damage detected perioperatively. Usually, the elbow is placed in 30–45° elbow flexion with the forearm in a neutral position, and the wrist is often supported. Post-operative position is decided by the surgeon, based on tension and quality of the tendon repair. This position is unique to each patient. The goal is a progressive elbow flexion by weekly advancing the range of motion block. Also, in our series a few years ago, we used the RICE (rest, ice, compression, and elevation) protocol and left the cast splint for the first 2 to 3 weeks. This acronym was first used by Dr. Mirkin [27]. However, Mirkin himself later admitted that rest was not the best way to heal an injury. Ice packs for the first few days after surgery are really effective in controlling pain and helping to reduce swelling. Elevating the limb slightly above body level by supporting the brace acts as a preventive measure against the increase in swelling that occurs for the first 2 weeks in the forearm area, and mild compression prevents unwanted movements. Exercises that can be practiced in the first phase: passive rotation of the forearm, passive later active flexion of the forearm gradually up to 45°. It is not recommended to massage the surgical scar area. It is also important to focus on the PROM (Passive Range of Motion) in the shoulder joint area, which is stiffened when the brace is worn 24 h a day for several weeks, which has its own weight. The long head triceps tendon on the scapula also plays a role.

## 5. Phase 2

### 5.1. Histopathologist’s View: Phase 2—Proliferative

Tenocytes form new collagen fibers and gradually bridge the defect. The main component of the extracellular matrix (ECM) is type I collagen. Its structure, and thus strength, is not like that of mature collagen fibers, which have developed a precise spatial structure. How the spatial arrangement of collagen is gradually formed is not clear at present. Tendon stem/progenitor cells called TSPCs also come in and help to repair the damaged site. Transcription of specific genes—scleraxis (Scx), mohawk (Mkx), and Egr1, which induce tenogenesis in stem cells—occurs. Angiogenesis occurs immediately after injury, and the number of vessels/mm^3^ gradually increases with a peak on day 3, followed by a gradual decrease during phase 2.

### 5.2. Rehabilitation Doctor’s View: Phase 2—Restoration of Mobility

From the very first days after the operation, the patient performs active flexion in the elbow joint to the extent allowed by the surgeon’s adjustment of the dynamic brace. The main goal in phase 2 is to achieve full ROM (range of motion)—the act of moving as far as anatomically possible during a given exercise. In some situations, movements are sometimes restricted for several weeks in order to avoid gap formation between the sutured tendons, jeopardizing their healing. In this phase we can start with early controlled motion (ECM). There is not really a consensus in the literature about the optimal time frame to begin early controlled motion. Currently, we start the exercise phase immediately after suture removal, i.e., on post-operative day 8 to 12. This phase is crucial in terms of range of motion in the elbow joint. The goal is to achieve a normal range of motion—extension 0° to −10°, flexion up to 140–150° and pronation 90°. At the first meeting with the physiotherapist, the range of passive movement will be measured with a goniometer, grip strength will be measured with a dynamometer of both limbs, and the circumference of both limbs will be measured for comparison in the elbow, forearm, and just below the deltoid muscle (most pronounced atrophy in the shoulder area). Gradually increase the range of motion at both the elbow and shoulder—increasing the angle of the elbow ideally 10–15° per week. Painfulness as the tendon heals paradoxically subsides as it is gradually loaded. Leaving the brace on overnight is a prevention of damage to the suture in sleep. In general, it is advisable to exercise independently at least twice a day, and each visit to the rehabilitation clinic is associated with a change of exercises according to the improvement of the range of motion. We can use stick exercises—where the healthy hand provides passive movement at the injured limb—and ball exercises to exercise the forearm muscles. We provide ongoing skin care and massage of the surgical wound to prevent post-operative adhesions to the skin. Properly chosen load increases collagen repair; conversely, overloading or underloading can have negative effects on the tendon. Mechanical stimulation promotes cell proliferation and collagen synthesis, which improves tendon repair and remodeling, increases tendon tensile strength, and reduces adhesion. Mechanical loading in varying degrees has a positive effect during all phases. The combination of Phase I and Phase II takes approximately the first 6 weeks after surgery, and this is exactly the time it takes for the patient to reach full range of motion. Some centers also use supportive therapies such as low-frequency ultrasound [28], pulsed magnetic fields [29], and dry needle technique. Two to three sessions at weekly intervals are usually recommended; the effect occurs relatively quickly within 48 h and is associated with improved blood circulation, stimulation of cellular activity, and pain relief. The term “dry” is intended to emphasize that the needle does not serve to administer any medication [30]. Another method reported in the literature is, e.g., “band flossing”—a method that consists of fixing the affected joint with a special bandage that causes compression, thereby reducing pain during movement and, conversely, after removal, a “sponge effect” improves blood circulation to the affected area [31]. We do not indicate supportive therapy in the patient and rather emphasize exercises. Swimming exercises are recommended about 5 weeks after surgery. All other types of exercises are performed standing or seated, without much involvement of the triceps itself.

## 6. Phase 3

To achieve effective healing, a balance needs to be struck between too low a load, which leads to increased adhesions, slowed maturation of reparative tissue, and, in particular, stiffness and thus joint soreness, and too high a load, which leads to tearing or rupture at the original site of damage.

### 6.1. Histopathologist’s View: Stage 3—Remodelling

After about 10 weeks, the fibrous tissue gradually changes to scar tissue, and this process continues for the following years. Mature tendons contain predominantly tenocytes and tenoblasts, which account for around 90–95% of the cell population [32]. The healed tissue does not reach the biomechanical properties before injury, and abnormality can be observed even one year after injury [33]. Due to the large difference in mechanical properties between tendon and bone tissue, a large concentration of stress is generated at the junction of the two parts, and the rights of this junction are at the highest risk of damage during postoperative rehabilitation. The attachment of the uninjured tendon to the bone is the so-called “enthesis”. This site is exposed to particularly high mechanical forces, and protection from damage is addressed by the condensed structure of the tendon at the junction and the shallow angle of attachment of the tendon to the bone [34]. The extracellular matrix (ECM) remodels and forms a more organized structure through collagen exchange with the formation of collagen cross-links. Cell density and vascularity gradually decrease.

### 6.2. Physiotherapist’s View: Phase 3—Muscle Strengthening

Here comes the main tool for restoring the functional and biomechanical properties of the tendon to pre-injury levels, and that is gradual loading of the tendon. The tendon is a mechanosensitive tissue, and this property allows us to treat it when it is injured based on mechanical loading and deforming it sufficiently [35]. Gradual loading of the triceps muscle starts on an individual basis, usually after 6 to 10 weeks. Here, we have found it very useful to start with the exercise in the prone position, the bent elbow hanging loosely off the edge of the bed, and slowly the patient tries to extend the arm and thus actively engage the triceps muscle itself. Also, in this position it is excellent to practice the shoulder joint, but in a relaxed manner, not trying to forcefully tighten the range of motion. Here, the patient suddenly realizes how greatly the triceps muscle has weakened by its inactivity. At this time, our goal is to gain the strength back. Theories state that our muscles are able to handle 20–50% more load in the eccentric phase of the movement than in the concentric phase of the movement, which implies that we can take advantage of the higher load and create more mechanical tension in eccentric training. Mechanical tension is directly proportional to building muscle mass. This is a logical explanation for why many studies begin with eccentric exercises with the assistance of a second person or machine. Muscles do not use their full potential in the eccentric phase, which in turn happens in the concentric phase of the movement. In the eccentric phase of the movement, we perform a slower movement, with better focus on the range of motion itself, thus preventing injury. Eccentric training, on the other hand, is more demanding for recovery, and therefore we do not perform strength exercises every day. In our case, however, with atrophied muscle, the mere initiation of strength training is such a significant boost that muscle mass grows very quickly in the beginning, regardless of exercise technique. This phase has the main motto: injury healing is driven by applied mechanical loads. Remodeling of the scar tissue itself in the injured area takes at least one year. An example for an eccentric contraction of the triceps, i.e., from the outstretched arm, we go to the elbow flexion by the so-called braking force—leaning both outstretched arms against the wall and gradually bringing the head closer to the wall, or on the pulley, gradually releasing the weight upwards [36]. When exercising with weights, the principle of small weights with a large number of repetitions applies. Characteristically, and usually surprisingly to the patient, the volume of the muscle itself lags visually despite the increase in muscle strength. Usually sixteen weeks after the operation, sports-specific and work-specific activities begin. On the other hand, high loads at the suture site can lead to gaps, microtears, or rupture of the suture site and consequently to poor healing. Of course, we must never exercise against pain during any of the exercises.

## 7. Materials and Methods

Twenty-three patients with triceps ruptures treated by surgery were included in the retrospective study. All procedures for this retrospective study were approved by our Institutional Review Board for this retrospective clinical study. All procedures followed were in accordance with the ethical standards of the responsible committee on human experimentation (institutional and national) and with the Helsinki Declaration of 1975, as revised in 2008. All patients underwent proper clinical examinations, including pre-operatively standardized MRI and, in the majority of cases, ultrasound scanning and then treated by surgery. Physical examination included the assessment of pain at the affected area, a questionnaire for elbow weakness and functionality assessment, and palpation for a palpable defect. Standardized ultrasound scanning examination (ultrasonography) was performed by an experienced ultrasonographer under the supervision of a musculoskeletal fellowship-trained radiologist. Also, MRIs images were read by fellowship-trained musculoskeletal radiologists. The surgical technique does not influence the length of the postoperative follow-up period. The final obligatory follow-up was conducted upon the conclusion of the third phase of rehabilitation. The median follow-up period was 4.2 months, with an interquartile range of 3.3 to 5.4 months. This indicates that the majority of patients have a comparable length of follow-up.

The University Hospital in Bratislava (the capital of the Slovak Republic) serves a catchment area of nearly 500,000 inhabitants and is the largest hospital in the Slovak Republic. This information helps to give an approximate idea of the prevalence of triceps tendon injuries in this region. Between 2014 and 2024, over the past 10 years, 23 patients with triceps tendon injuries were treated at the emergency or surgical outpatient clinic of this hospital. Four distinct types of injuries were identified: acute tears, chronic tears, partial tears (either acute or chronic), and snapping triceps. The study excluded patients under 18 years of age, those with open injuries in the elbow area, patients with associated fractures in the elbow joint, patients with snapping triceps, and patients with chronic ruptures where primary tendon repair was not possible. The data were obtained from hospital records. The issue of obtaining informed consent prior to surgical treatment, which in our hospital always encompasses the potential utilization of documentation for scientific purposes, remains a highly contentious topic in the medical community. In establishing the doctor-patient relationship, it is crucial to recognize that the duty to provide information shifts from what “a reasonable doctor or practitioner” might consider essential for the patient to know to what “a reasonable patient” would need to know in order to make an informed decision. All legal systems in the European Union require informed consent to be obtained before any medical procedure is carried out, and there is a clear regulatory framework. The information and subsequent expression of consent are based on a fiduciary relationship between the doctor and the patient. In Europe today, the requirement for safety and quality of healthcare has long since replaced the ‘original’ requirement for health, which is fully linked to the direct participation of users in life decisions [37]. In our subgroup of patients, we focused on the patient’s ability to engage in informed decision-making in relation to their healthcare. We paid particular attention to the overall benefit and appropriateness of the care provided to ensure that it was tailored to the patient’s individual needs. It is essential that patients receive clear and comprehensive information regarding their treatment, available therapeutic options, and potential risks, including in the post-operative period. In the Slovak Republic, only the doctor is responsible for providing instructions to the patient. The question of the actual surgical technique and its alternatives is explicitly delineated in the written consent form, which is provided to the patient prior to the surgical procedure. In this document, the patient confirms in writing that they are aware of the three basic surgical techniques that may be employed and that they consent to the surgeon’s discretion in selecting one of these techniques based on the findings of the perioperative evaluation. Conversely, other factors such as the timing of surgery, the maximum waiting period, and the potential for alternative settings, including conservative management, are seldom taken into account, despite their relevance to the patient. It is reasonable to conclude that each health professional is responsible for their own actions that may affect psychophysical integrity and should therefore be required to inform and obtain consent individually. However, when considering consent to treatment in a broader sense, it is an error to break down the organic process into hundreds of micro-activities carried out by individual, isolated professionals with a single act of consent, particularly when this needs to be documented in writing. Out of the 23 cases, based on clinical findings and imaging studies, surgical treatment was recommended for 12 patients. Two patients declined surgery due to their subjective perception of sufficient range of motion, age, and the nature of their work. We conducted this retrospective cohort study on 10 patients with DTTR between 2014 and 2024. The procedure was performed under a supraclavicular block or general anesthesia. The patient was positioned either in the lateral decubitus or prone position, and the use of a tourniquet was left to the surgeon’s discretion. A 10 cm posterior incision was made. The surgeon could repair the distal triceps rupture using the technique they deemed appropriate or were most experienced with, as no single method appeared to offer a lower risk of complications or reoperation.

For the data analysis we used Microsoft IBM SPSS software—version 25. 

## 8. Results

Based on the processed data, we can formulate the following conclusions.

1. Incidence

Our findings align with the literature, confirming that, unlike tendon injuries in other locations where incidence is rising for various reasons, triceps tendon ruptures remain a rare injury. University Hospital provides healthcare services in the capital of Slovakia, which has a population of approximately 500,000. Over a 10-year period, we treated 23 patients, averaging 2.3 cases per year. This incidence in our region is 0.46 cases per 100,000 inhabitants. For purposes of comparison, the estimated national incidence of distal biceps tendon rupture is 2.55 per 100,000 patient-years [38].

2. Evaluation of demographic data

**Figure 6:**
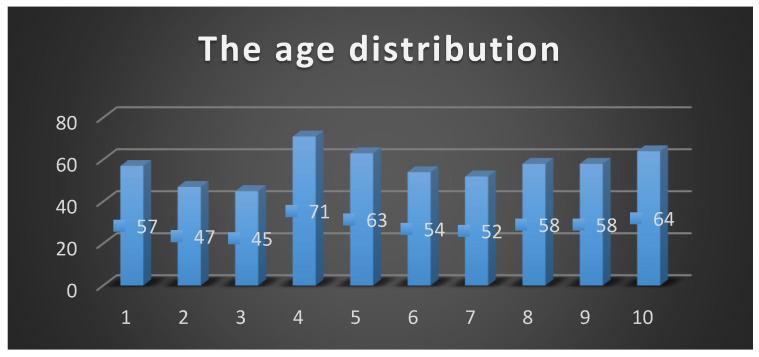
Proportion of unoperated patients with a verified partial triceps lesion.

Demographics: Triceps tendon ruptures most commonly affect men in the fourth and fifth decades of life. From our pooled results we observed the mean age at rupture to be 57.7 years. There was a 9:1 ratio of males to females (Figure 6).

3. The indication for surgery

Over a 10-year period, 23 patients with confirmed triceps tendon injuries were treated. When determining the indication for surgery, we consider multiple factors (see above). The decisive factor is assessing the extent of tendon damage. As mentioned earlier, any tear involving more than 50% of the tendon’s integrity, as well as full-thickness tears, are managed surgically. In borderline cases, MRI plays a key role in the decision-making process. Although this imaging is not available 24/7 in our facility, it can be performed within 24 h for such cases. In our patient group with partial or complete tendon lesions, fewer than 50% were indicated for surgical intervention.

4. Qualitative indicator of health care

**Figure 7:**
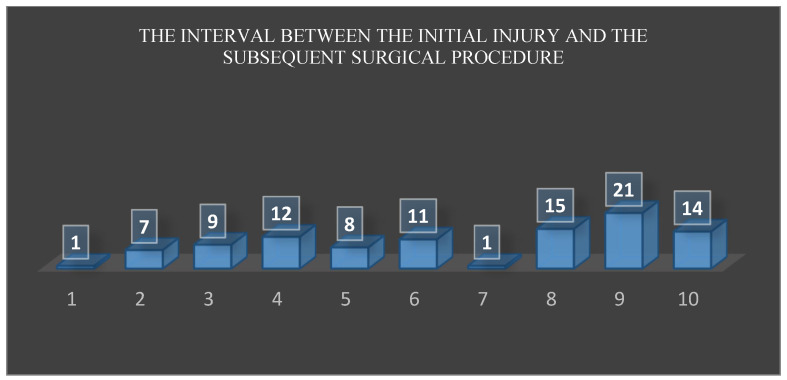
The interval between the initial injury and the subsequent surgical procedure.

In cases of partial tendon rupture, an additional MRI examination is necessary to assess the extent of the damage. Additionally, in patients with comorbidities, especially since these injuries typically affect older individuals, further examinations are required as part of the standard preoperative workup for semi-urgent surgeries. On the other hand, the time between the injury and surgery is crucial for the success of the primary repair. An acute rupture is defined as an injury occurring within 3 weeks of trauma. For chronic ruptures, primary tendon repair is usually not possible, and tendon transfers are required. In our patient group, no tendon transfers were necessary as all surgeries were performed within 3 weeks of injury. According to the literature, the median time from triceps rupture to surgery is 22 days [39]. In our patient group, the time from injury to surgery was from 1 to 21 days (Figure 7).

5. Preoperative magnetic resonance examination and ultrasound

The first application of ultrasound (US) was performed during World War I for the detection of submarines (sonar) [40]. The main advantages of diagnostic US in medicine include real-time assessment, absence of radiation, reduced cost, and portability [41]. It is erroneous to consider ultrasound as a rival imaging modality; rather, it should be regarded as a supplementary imaging modality. In order to undertake an ultrasound (US) examination of the triceps tendon, it is essential to have direct experience of performing US examinations of the tendon. In this location, due to the more complex anatomical proportions at the site of interest, the phenomenon of anisotropy observed during ultrasound imaging becomes fully apparent. This is a change in the resulting echo of the tendon when the angle of the transducer is changed. If the tendon fiber has a light appearance and the transducer is positioned perpendicular to the tendon, it may appear darker. Conversely, if the transducer is oriented obliquely, it can potentially introduce significant errors. The significance of lesion localization in determining the most appropriate examination method is substantiated by the preference for ultrasound over MRI in the assessment of the rotator cuff. One meta-analysis of rotator cuff imaging has shown that ultrasound has an accuracy of 95% [42]. Dynamic sonography is also instrumental in differentiating partial and full-body tendon tears. The absence of tendon translation through the affected area and retraction of the torn tendon stump are indicative of a full-body tear. Conversely, these techniques are only applicable to the evaluation of an isolated tendon, rendering them ineffective in assessing tendons that converge with other tendons. In conclusion, ultrasound can be regarded as a viable alternative to MRI in instances where MRI is contraindicated or when the patient is unable to tolerate MRI. The findings of all studies comparing MRI and US indicate that MRI is a more precise imaging technique for identifying the type of tear, although US is a more cost-effective option [43]. When using an MRI as a decisive factor in determining the indication for surgery in unclear cases, we were also interested in the percentage of MRI examinations performed pre-operatively. Unfortunately, this number is skewed, as it is not possible to accurately determine for non-operated patients. These patients typically continue treatment and follow-up in outpatient clinics and private rehabilitation facilities outside of our hospital.

6. Technique of surgical procedure

**Figure 8:**
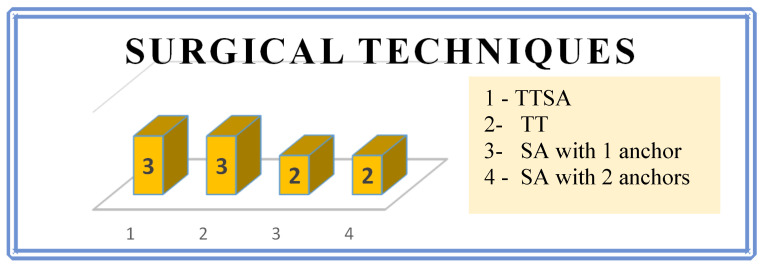
The surgical techniques employed in the patient cohort can be broadly classified into four categories. TTSA—transosseous tunnel plus suture anchor repair; TT—suture-only transosseous tunnel repair. SA—suture anchor repair with one or two anchors.

Recently, instrumentation for tendon fixation has significantly become widespread. An ideal suture anchor is easy to handle, maintains sufficient pull-out strength, prevents suture abrasion, and is absorbable without resulting in any reactions as the material dissolves [44]. Currently, biodegradable suture anchors have been developed to help overcome complications associated with metallic anchors [45]. It is essential that surgeons understand key characteristics of a variety of currently available anchors [46]. In our series, all three standard methods of fixation of the triceps tendon to the olecranon were used, and none of the methods is currently dominant. There were 12 surgical procedures performed in 10 patients with triceps tendon lesions. In one patient, two surgical revisions were required because of infection in the surgical wound. Due to the rarity of this occurrence, a special team was not formed to deal with this problem, and patients were operated on by different surgeons. There is no consensus among our surgeons on the best method of repair. The procedure was completed under a supraclavicular block. Approximately 10 cm posterior incision was made (Figure 8).

7. Postoperative complications

The three most common complications with this type of injury include ulnar neuropathies, infections, and pain. These complications are detected in the immediate post-operative period and are therefore accurately evaluated in our series. In our series, one patient was found to have an infection in the wound. Subsequently, the condition was managed with 2 reoperations—the first involved revision, debridement, and loading of a vacuum drain, which we use for secondary wound healing, and then after 7 days the vacuum drain was removed, and a secondary wound suture was performed. The re-rupture rate remains problematic as it can occur several months after the injury, and patients with this injury are not usually dispensed, and efforts to follow up with patients have been unsuccessful. The re-rupture rate is reported to be relatively low, up to 4%, and in our series, it was not necessary to reoperate the patient for this reason.

## 9. Discussion

This comprehensive study on triceps tendon ruptures conducted over a period of a decade provides several important insights into the nature and treatment of this relatively rare injury. First and foremost, triceps tendon ruptures are infrequent, with only 23 cases treated over the course of 10 years, which averages out to 2.3 cases annually. This equates to an incidence rate of approximately 0.46 per 100,000 people in the capital of Slovakia, which underscores the rarity of this type of injury. The data also reveal that triceps tendon ruptures predominantly affect men between the ages of 40 and 50, with a striking male-to-female ratio of 9:1. The mean age of rupture in the cohort was found to be 57.7 years, further indicating that this injury is more common in middle-aged individuals.

Surgical intervention is the preferred treatment option, particularly in cases where the tendon rupture is extensive—defined as tears involving more than 50% of the tendon or full-thickness ruptures. In many instances, MRI plays a critical role in evaluating partial tears or borderline cases, guiding the decision-making process for surgery. Prompt surgical intervention is crucial, as delays beyond three weeks post-injury are associated with an increased risk of complications related to chronic rupture, such as muscle atrophy and decreased function. Encouragingly, all patients in this study underwent surgery within 21 days of their injury, minimizing the risk of such complications. MRI imaging is especially helpful in assessing the extent of injury in cases of partial tears and is also instrumental in managing patients with comorbid conditions that may complicate recovery.

Advances in surgical techniques, particularly the introduction of biodegradable suture anchors, have improved fixation methods and the overall success of surgeries. Despite this progress, there is still no consensus on a single dominant surgical method, as surgeons often choose techniques based on their individual experience and the specifics of the injury. Common postoperative complications observed in this study include ulnar neuropathies, infections, and persistent pain. However, it is worth noting that only one patient required revision surgery due to an infection, which was managed with a vacuum drain. Although re-ruptures remain a concern, follow-up is often challenging due to the relatively long recovery times associated with these injuries, and no cases of re-rupture required reoperation in this cohort.

The findings of this study highlight the importance of timely and effective treatment for triceps tendon ruptures and the ongoing advancements in surgical materials and techniques. The results also align with previous studies, such as that of Koplas et al. [5], which reported a prevalence of triceps tendon injuries of 3.8%. In their study, 5 women and 23 men were found to have either partial or complete tears, with the mean age of those affected being 46.6 years, a finding similar to ours. Additionally, the gender distribution observed in their study mirrors our own, with middle-aged men being the most commonly affected group. Koplas et al. also noted that triceps brachii ruptures often go clinically undiagnosed, a point that aligns with our own observations.

Supporting our findings, Giannicola et al. [47] confirm that distal triceps tendon ruptures (DTTR) are indeed a rare injury. Anzel et al., in their analysis of 1014 tendon ruptures, reported a prevalence of 0.8% for DTTR. More recent studies, including Giannicola’s, have shown a higher prevalence of triceps tendon tears, citing 3.8% of cases found through MRI investigations. Despite these numbers, it remains highly likely that a significant proportion of patients with this injury go undiagnosed. Several factors contribute to this, including the clinical condition immediately following the injury. For instance, if the lateral extension and the functional anconeus remain intact, active elbow extension may still be preserved, making the diagnosis less obvious. Additionally, the swelling and pain that accompany the injury limit the availability of imaging and investigative options. The rarity of the injury itself means that clinicians may be less inclined to consider it as a possibility, further contributing to the likelihood of an undiagnosed rupture.

Another challenge in managing triceps tendon ruptures is the relatively long healing time, which often extends up to a year before patients can return to pre-operative levels of muscle strength. This underscores the potential of telemedicine to enhance the quality of post-operative care in this context. Telemedicine is not a comprehensive substitute for conventional clinical examination. However, it does facilitate the monitoring of patients over extended distances and over prolonged periods, thereby alleviating discomfort. Once the application has been installed on the patient’s mobile device, communication with healthcare professionals can commence on a daily basis. At the present time, the potential of telemedicine is still not being fully realized in Europe. That is mostly due to the still high costs of setting up and performing telemedicine services. Also, the limited scope of implementation can be attributed to the deficiencies in the technical infrastructure in EU Member States, as well as concerns pertaining to confidentiality and privacy in the context of health data management [48]. 

This protracted recovery period complicates both diagnosis and treatment, as the injury may not become apparent until the muscle strength does not recover as expected. Eighteen different scoring systems are currently available to evaluate elbow disorders, each with its own set of objective and subjective criteria. However, there is no single, universally accepted outcome evaluation system that is both reliable and sensitive enough to detect clinically relevant changes. The variability in patient groups, imaging techniques, and reported outcomes makes it difficult to draw reliable conclusions across different studies, highlighting the need for more consistent and standardized approaches to assessing and treating triceps tendon injuries.

## 10. Conclusions

Triceps tendon ruptures occurrence is rare, with only 23 cases over 10 years, averaging 2.3 cases per year, equating to an incidence rate of 0.46 per 100,000 people in Slovakia’s capital. The injury predominantly affects men aged 40–50, with a 9:1 male-to-female ratio, and the mean age of rupture was 57.7 years. Surgical intervention is primarily considered for tears involving more than 50% of the tendon or full-thickness ruptures, with MRI often guiding borderline cases.

Adult tendons have a limited natural healing capacity, which derives from the nature of the tendon with its poor cellularity, limited vascularization, and low metabolism. Tendon tissue heals by fibrovascular scarring, and current treatment strategies fail to restore the functional, structural, and biochemical properties of the tendon to the level of the original tissue [49,50,51]. Currently, despite new knowledge gained from studies in animal models and technological advances, tendon tissue restitution and repair remain an ongoing problem [51]. In the near future, randomized trials will have to be designed in order to better handle intermediate results and, most importantly, to evaluate long-term follow-up as well.

## Data Availability

Due to privacy and ethical reasons we do not wish to share additional data.

## Abbreviations

TT	suture only transosseous tunnel repair
SA	suture anchor-only repair
TTSA	transosseous tunnel plus suture anchor repair
DTTR	distal triceps tendon ruptures
PROM	passive range of motion
